# Resistance to Thyroid Hormone Beta and Coexisting Thyroid Disease: Diagnostic and Therapeutic Challenges Illustrated by Two Cases

**DOI:** 10.1155/crie/9523606

**Published:** 2025-10-21

**Authors:** Sara Ribeiro, Ana Varela, Joana Queirós

**Affiliations:** ^1^Department of Endocrinology, Diabetes and Metabolism, São João Local Health Unit, Porto, Portugal; ^2^Faculty of Medicine, University of Porto, Porto, Portugal

**Keywords:** amiodarone-induced hypothyroidism, Hashimoto's thyroiditis, individualized treatment, resistance to thyroid hormone (RTH), thyroid dysfunction

## Abstract

Resistance to thyroid hormone (RTH) is a rare clinical syndrome characterized by reduced tissue responsiveness to thyroid hormone (TH), typically presenting with elevated TH levels without suppression of thyrotropin (TSH). In most cases, RTH is caused by mutations in the TH receptor beta (*THRB*) gene. While treatment is generally unnecessary due to preserved endogenous compensation, this physiological balance may be disrupted in the presence of compromised thyroidal reserve. We report two unrelated female patients with genetically confirmed RTH and coexisting thyroid disease. The first, an 18-year-old with Hashimoto's thyroiditis, required unusually high doses of levothyroxine to maintain TSH within the normal range and was later diagnosed with RTHβ. The second, a 54-year-old with known RTH, developed tachyarrhythmia and amiodarone-induced hypothyroidism, complicating TH replacement. In both cases, the coexistence of RTH and acquired thyroid disease obscured the clinical picture and posed significant therapeutic challenges. These cases illustrate how superimposed thyroid pathology can destabilize the typically compensated state of RTH, underscoring the importance of maintaining a high index of suspicion in patients with persistent, unexplained thyroid function abnormalities. Early recognition, personalized management, and lifelong follow-up are essential to ensure optimal outcomes and avoid unnecessary interventions.

## 1. Introduction

The physiological actions of thyroid hormones (THs) are mediated by two nuclear receptors—thyroid hormone receptor alpha (TRα) and beta (TRβ)—encoded by *THRA* and TH receptor beta (*THRB*) genes, respectively. These receptors regulate gene expression in target tissues by binding to thyroid response elements in DNA, usually as heterodimers with the retinoid X receptor (RXR). Upon free triiodothyronine (FT3) binding, a conformational change induces the release of corepressors and recruitment of coactivators, activating the transcription of TH-responsive genes [1].

Resistance to TH (RTH) is a rare inherited syndrome characterized by reduced tissue responsiveness to TH, most commonly caused by mutations in the *THRβ* gene [2, 3]. The condition—referred to as RTHβ—has an estimated prevalence of 1 in 20,000 to 1 in 40,000 live births, though it is likely underdiagnosed due to clinical heterogeneity [3]. RTHβ is typically inherited in an autosomal dominant pattern, with mutant TRβ receptors exerting a dominant-negative effect on normal receptor function, impairing the transcription of T3-regulated genes [4].

THRβ is predominantly expressed in the hypothalamus, pituitary, liver, and kidney. Impaired TH sensitivity in the hypothalamus and pituitary disrupts the negative feedback, resulting in inappropriately normal or elevated thyrotropin (TSH) levels despite increased free thyroxine (FT4) and FT3 concentrations [5]. In most individuals, a compensatory increase in endogenous hormone production preserves a euthyroid state at the tissue level. Nevertheless, clinical expression is highly variable—ranging from asymptomatic individuals to those with signs of thyrotoxicosis (tachycardia, advanced bone age, hyperactivity), hypothyroidism (hypercholesterolemia, impaired cognitive ability), or both, depending on tissue-specific receptor distribution and expression. Goiter is the most common finding, reported in up to 95% of cases [3, 6]. Sinus tachycardia is also very common and, together with goiter, often leads to the erroneous diagnosis of autoimmune thyrotoxicosis [7]. When the diagnosis is incorrect and antithyroid drugs are initiated to normalize FT4 and FT3 levels, symptoms may paradoxically worsen due to the underlying RTH—further reducing hormone action in target tissues despite “normal” circulating values.

In contrast, mutations in *THRA* are exceedingly rare and define a distinct clinical entity known as RTHα. TRα is mainly expressed in the brain, heart, gastrointestinal tract, and skeleton, and its deficiency results in a more severe and less compensated phenotype. Affected individuals may present with developmental delay, growth retardation, bradycardia, constipation, and other tissue-specific manifestations, often in the setting of near-normal thyroid function tests (TFTs) [8, 9].

Although RTHβ itself is uncommon, its coexistence with other thyroidal disorders—such as autoimmune thyroiditis or amiodarone-induced dysfunction—is exceptionally rare. These overlapping conditions can destabilize the usually well-compensated state of RTH and obscure the diagnosis when it is not yet recognized. Importantly, as illustrated in one of our cases, thyroidal decompensation may also occur in patients with a previously established diagnosis of RTH, particularly in the context of intercurrent illness or iatrogenic triggers. This underscores the importance of long-term follow-up, even after diagnosis, to anticipate and manage evolving clinical challenges.

## 2. Cases Presentation

### 2.1. Case 1—RTH Beta in a Patient With Autoimmune Hypothyroidism (AIH)

**Figure 1:**
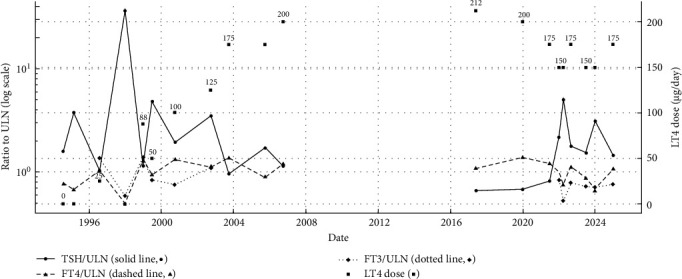
Thyroid function normalized to contemporaneous ULN over time (case 1). TSH/FT4/FT3 are plotted as ratios to the same-date upper limit of normal (ULN, normalizing across assays with different reference ranges); the *y*-axis is log-scaled to visualize proportional increases and decreases.

An 18-year-old female medical student was referred to the Endocrinology Clinic due to persistently elevated TSH despite long-term levothyroxine therapy. She had been diagnosed with autoimmune thyroiditis at the age of 7 years, following the development of a goiter. At that time, TFTs revealed an elevated TSH (8.75 µIU/mL), normal FT4 (1.31 ng/dL), and positive thyroid antibodies (Table 1 and Figure 1). Apart from AIH, her past medical history was unremarkable. Thyroid ultrasound demonstrated a diffusely enlarged gland with no nodules (measurements not available).

During follow-up in pediatric endocrinology, both the patient and her mother consistently reported good adherence to levothyroxine therapy. However, TSH levels remained elevated, requiring frequent dose adjustments.

At her initial adult endocrinology evaluation, she weighed 55 kg and was 1.65 m tall (BMI: 20.2 kg/m^2^). Her heart rate was normal, and the physical examination was otherwise unremarkable except for a visibly enlarged thyroid. She was taking a high dose of levothyroxine (200 mcg/day; approximately 3.6 mcg/kg/day). TFTs continued to show elevated TSH with FT4 levels at the upper limit of normal. Thyroid ultrasound showed a heterogeneous, hypoechoic, and enlarged gland (the right lobe measuring 6.2 cm × 2.5 cm × 2.0 cm and the left lobe 5.5 cm × 2.0 cm × 1.8 cm).

Over the subsequent years, TSH levels remained difficult to normalize. Normalization was only achieved when FT4 levels were above the normal reference range, at times associated with clinical symptoms of thyrotoxicosis (e.g., weight loss and tremor). These findings prompted a reevaluation of the initial diagnosis in 2022. Repeat antibody testing confirmed persistently elevated antithyroid peroxidase (anti-TPO) and anti-thyroglobulin antibodies. Given the high-dose levothyroxine requirement and persistently elevated TSH levels despite normal to high-normal FT4, RTHβ was suspected, and genetic testing was performed. Targeted sequencing identified a heterozygous *THRB* variant (NM_000461.4:c.1292T > C; p.(Ile431Thr)), supporting the clinical diagnosis of RTHβ.

**Figure 2:**
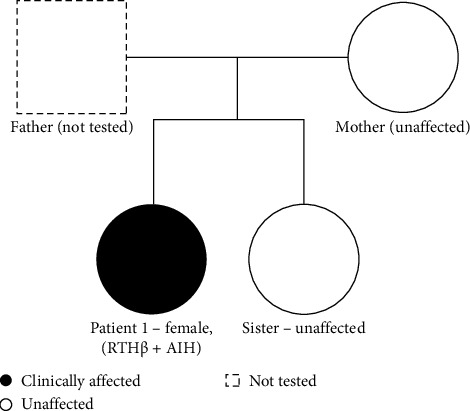
Family pedigree (Case 1).

This case illustrates diagnostic challenges arising from the coexistence of AIH and RTH. The absence of pre-treatment biochemical values partially obscured the typical RTHβ biochemical signature and delayed recognition of the underlying disorder. Her mother and sister had normal thyroid function and tested negative for the familial *THRB* variant, and the father was unavailable for testing (Figure 2). At her most recent follow-up, ultrasound revealed a small, markedly heterogeneous thyroid gland, reflecting the chronic evolution of autoimmune thyroiditis.

She continues on individualized levothyroxine therapy, with titration based on both clinical status (heart rate, blood pressure, weight change, tremor, sleep disturbance, and menstrual cycles) and biochemical parameters (TSH, FT4, FT3, and lipid profile). Additional objective monitoring included bone mineral density and periodic electrocardiogram (ECG). The current therapeutic goal is to maintain a balance between slightly elevated TSH (as the patient seems to develop symptoms of thyrotoxicosis when a normal TSH is achieved) and mildly elevated FT4 levels, optimizing her functional status while avoiding overtreatment. Of note, despite elevated FT4 levels, no sustained tachycardia was documented, and heart rate remained within the normal range throughout follow-up.

### 2.2. Case 2—Amiodarone-Induced Hypothyroidism in a Patient With RTHβ

A 54-year-old female was referred for evaluation of a thyroid nodule. Her medical history was notable for difficult-to-control asthma and a hypereosinophilic syndrome of undetermined etiology, for which she was under regular follow-up in internal medicine and pulmonology. At initial presentation, she was asymptomatic, and the thyroid was not palpable on physical examination.

**Figure 3:**
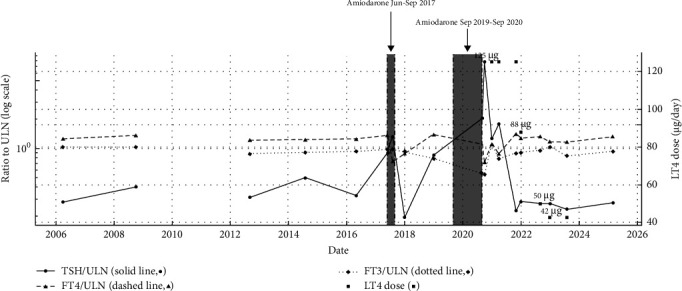
Thyroid function normalized to contemporaneous ULN over time (Case 2). TSH/FT4/FT3 are plotted as ratios to the same-date upper limit of normal (ULN, normalizing across assays with different reference ranges); the *y*-axis is log-scaled to visualize proportional increases and decreases.

**Figure 4:**
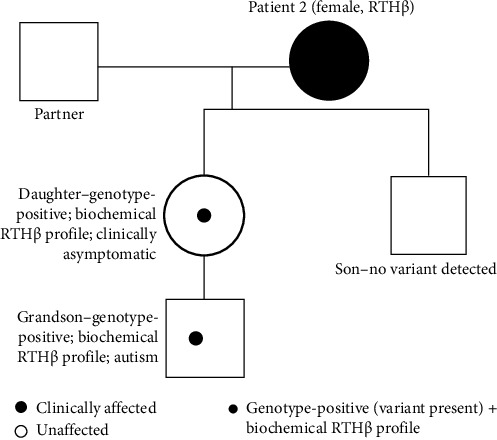
Family pedigree (Case 2).

Initial TFTs revealed elevated free T3 (FT3:3.86 μg/dL; reference range: 1.75–3.75) and free T4 (FT4:1.86 ng/dL; reference range: 0.70–1.48), with a normal TSH (1.55 μIU/mL; reference range: 0.35–5.50) (Table 2 and Figure 3). Thyroid scintigraphy showed diffuse hypercaptation, and cervical ultrasound demonstrated a globose thyroid with overall dimensions within the normal range (right lobe 4.3 cm × 2.1 cm × 1.4 cm; left lobe 4.3 cm × 1.8 cm × 1.3 cm). A single 8 mm nodule was identified in the posterior aspect of the right lobe, classified as ACR TI-RADS 3. A TRH stimulation test demonstrated an appropriate TSH response. Biochemical findings remained consistent on subsequent assessments. Genetic testing confirmed a diagnosis of RTHβ, identifying a heterozygous pathogenic variant in the *THRB* gene ((NM_000461.4):c.1707C >G; p.(Pro453Ala). Family screening revealed the same variant in one clinically asymptomatic daughter and a grandson with autism, both showing the characteristic biochemical RTHβ profile (elevated FT4/FT3 with nonsuppressed TSH) (Figure 4).

The patient remained clinically euthyroid and untreated for several years, with persistently elevated FT4, variable FT3, and consistently normal TSH values. During this period, she was monitored with regular blood pressure measurements and occasional ECGs. As she remained asymptomatic, no further cardiological surveillance was performed.

Six years into follow-up, she developed a supraventricular tachyarrhythmia and was diagnosed, at another hospital, with antidromic atrioventricular (AV) conduction with a functional bundle branch block and rapid ventricular response. She underwent successful catheter ablation and was started on bisoprolol (5 mg/day), with treatment adherence consistently confirmed throughout follow-up.

Five years later, she experienced a recurrence requiring both pharmacological and electrical cardioversions. Amiodarone was reinitiated by the cardiology team, resulting in a transient elevation in TSH (7.28 μIU/mL) and a drop in FT4 to the normal range (1.06 ng/dL). Amiodarone was discontinued shortly thereafter, with a gradual return of thyroid function to her baseline profile. She remained on bisoprolol and warfarin for atrial fibrillation (AF).

Seven years after the initial arrhythmia episode, she was admitted to the intensive care unit (ICU) with cardiogenic shock secondary to tachycardia-induced cardiomyopathy. Cardioversion and attempted AV nodal ablation were unsuccessful, prompting pacemaker implantation and reinitiation of amiodarone.

Ten months later, she was readmitted for decompensated heart failure with a reduced ejection fraction (EF 19%). TFTs at that time showed markedly elevated TSH (42.7 μIU/mL), with free T3 (2.02 μg/dL) and free T4 (1.06 ng/dL) within or near the reference range. At this time, the patient reported fatigue and exertional intolerance, with no additional classical hypothyroid symptoms such as cold intolerance, weight gain, and constipation. Thyroid antibodies were negative. Although the free hormone levels were not overtly low relative to the normal reference ranges, the significantly elevated TSH, along with decreased FT3 and FT4 compared to her baseline levels, in conjunction with the clinical context, was consistent with amiodarone-induced hypothyroidism. Amiodarone was withdrawn as per cardiology recommendation, and levothyroxine therapy was initiated. At this stage, the therapeutic goal was defined as restoring TFTs to the patient's individual baseline, preventing hypothyroidism symptoms while minimizing the risk of cardiac decompensation. Treatment adequacy was monitored through serial cardiology evaluations (including Holter monitoring and echocardiography), lipid profile, and bone mass densitometry.

Over the following years, she remained clinically stable from a cardiology perspective, with significant recovery of left ventricular function. She was also diagnosed with osteoporosis, which is being managed accordingly.

Four years after starting levothyroxine, the medication was successfully discontinued. The patient currently maintains a normal TSH (1.52 μIU/mL) and a slightly elevated FT4, mirroring her pre-amiodarone baseline.

## 3. Discussion

A growing body of evidence suggests a higher risk of autoimmune thyroiditis in patients with RTHβ [10, 11];, although progression to overt hypothyroidism remains poorly defined. Only a limited number of cases describe the coexistence of AIH and RTHβ, complicating both diagnosis and treatment [12–14].

Fukata et al. [12] described a woman who was initially misdiagnosed with thyrotoxicosis and treated accordingly before a correct diagnosis of RTHβ was established. Overtime, she developed fatigue, goiter, and tested positive for antimicrosomal antibodies. As her TSH levels rose and FT4 remained within the normal range, she eventually required levothyroxine to reestablish her prior biochemical profile—namely, a normal TSH and elevated FT4—consistent with her RTHβ baseline [12].

Tran H.A. reported a 45-year-old woman with suspected RTHβ and coexisting AIH, who required unusually high doses of levothyroxine to achieve biochemical control. This case highlights the therapeutic challenges encountered when premorbid thyroid function is unknown, making dose titration and treatment targets difficult to define [13].

Similarly, Robinson et al. [14] described a patient with genetically confirmed RTHβ who progressed to symptomatic hypothyroidism. Initially clinically euthyroid with mildly elevated TSH, she later developed AIH. Despite FT4 levels remaining within the reference range, high-dose levothyroxine was necessary to restore her previous TSH values [14].

In contrast, Wu et al. [15] presented a 25-year-old woman with RTHβ who developed postpartum thyroiditis, with spontaneous return to her baseline thyroid function and no progression to permanent hypothyroidism. This case illustrates that transient deviations from baseline thyroid function may not require long-term treatment, reinforcing the importance of longitudinal follow-up to distinguish between temporary and sustained dysfunction.

Collectively, these reports underscore the need to interpret TFTs in RTHβ within the broader clinical context. When autoimmune thyroiditis is suspected or confirmed, deviations from the individual's established biochemical baseline, emergence of symptoms, and the presence of thyroid antibodies should raise concern for evolving hypothyroidism.

Our first case exemplifies these diagnostic challenges, highlighting the difficulties in interpreting biochemical profiles without a known premorbid baseline. Diagnosed with AIH in childhood, the patient presented with persistently elevated TSH despite reportedly high adherence and escalating levothyroxine doses. Targeted sequencing subsequently identified a heterozygous *THRB* variant (NM_000461.4:c.1292T > C, p.(Ile431Thr))—a rare missense change within the ligand-binding domain that, although listed in public databases as a variant of uncertain significance, is consistent with RTHβ in this clinical context and supports the clinical diagnosis. In retrospect, the patient biochemical profile—normalization of TSH levels only when FT4 concentrations were above the reference range—was highly suggestive of the underlying RTHβ. However, the coexistence of AIH made the pattern more difficult to recognize, complicating interpretation and ultimately delaying the diagnosis.

Importantly, patients with coexisting RTHβ and AIH often require higher-than-expected levothyroxine doses, and normalization of TSH may only occur with supraphysiological FT4 and FT3 levels—a pattern that may mislead clinicians toward erroneous assumptions of noncompliance or malabsorption. Our patient exemplified this pattern, highlighting the need to consider RTHβ in young individuals with unexplained TSH elevation and excessive hormone requirements.

It has been recommended by recent guidelines and consensus statements from the European Thyroid Association (ETA) and the Japan Thyroid Association (JTA), respectively, that levothyroxine treatment in patients with coexistent RTHβ and AIH should either be titrated to achieve FT4 comparable to other family members with RTHβ alone, titrated to maintain high-normal TSH and high free TH concentrations, or to achieve symptomatic improvement [16, 17]. Of note, these recommendations remain largely based on case reports, and further research is needed to fully determine the optimal treatment approach in this group of patients. In this case, without a pre-autoimmune baseline or known family members to compare, individualized dose adjustment was particularly challenging. Importantly, in this patient, well-being was achieved only with slightly elevated TSH and mildly elevated FT4, as full TSH normalization triggered thyrotoxic symptoms of thyrotoxicosis such as weight loss and tremors, highlighting the clinical variability of RTHβ expression.

Although most RTHβ patients remain clinically euthyroid and do not require treatment, intervention may become necessary when hypothyroidism arises from autoimmune or iatrogenic causes. In these settings, TH replacement must balance restoration of function with the risk of tissue-level hyperthyroidism (e.g., cardiac). Prolonged under-replacement and chronic elevation of TSH may also increase the risk of pituitary thyrotroph hyperplasia [17].

This principle guided the management of our second patient. With genetically confirmed RTHβ, she developed amiodarone-induced hypothyroidism following the onset of supraventricular arrhythmia. Amiodarone affects thyroid physiology through both its high iodine content and direct interference with TH metabolism [18], potentially leading to hypothyroidism or thyrotoxicosis Importantly, in patients with RTHβ—who typically present with elevated TH levels—the main concern is that amiodarone-induced hyperthyroidism or thyrotoxicosis may further increase circulating TH concentrations and cardiovascular risk. Conversely, hypothyroidism may also pose challenges in these patients, since treatment goals are difficult to define and supraphysiological hormone levels are often required to maintain well-being. This underscores that amiodarone may not always represent the optimal antiarrhythmic choice in individuals with RTHβ, and highlights the importance of a multidisciplinary discussion—particularly between cardiology and endocrinology—when selecting long-term rhythm control strategies. In our case, reintroduction of amiodarone for arrhythmia control led to persistent hypothyroidism, requiring levothyroxine therapy, which was gradually tapered and eventually discontinued a few years later. While AIH is more common and persistent in patients with underlying autoimmune thyroiditis [18], it remains unclear whether RTHβ itself increases susceptibility to amiodarone-induced thyroid dysfunction.

Documented associations between amiodarone and RTHβ are exceptionally rare. Alkundi and Momoh reported a similar case in which amiodarone exposure led to transient hypothyroidism and ultimately the diagnosis of RTHβ, although without the need for hormone replacement [19].

Thyrotoxic cardiac manifestations—mediated by elevated TH action on TRα-expressing myocardium—include tachycardia, AF, and tachycardia-induced cardiomyopathy [20–22]. These mechanisms likely contribute to the increased risk of adverse cardiovascular outcomes and earlier mortality described in RTHβ patients [23, 24]. In our case, supraphysiological TH levels in a myocardium expressing intact TRα may have exacerbated arrhythmia and contributed to decompensated heart failure. Although these complications may not be genotype-dependent [20, 21], elevated circulating TH levels can increase cardiac risk in predisposed individuals. Notably, our patient's daughter, who also carries the mutation, remains asymptomatic from a cardiovascular standpoint. Interestingly, one episode of heart failure decompensation in our patient occurred while she was biochemically hypothyroid due to amiodarone-induced thyroiditis, suggesting that preexisting structural or electrical heart disease may modulate the clinical impact of both hypo- and hyperthyroid states.

Recognizing the increased cardiovascular risk in RTHβ, “ETA Guidelines on diagnosis and management of TH transport, metabolism, and action” recommend risk assessment for all patients older than 30 years—or younger if symptomatic—and ongoing surveillance that should include systematic cardiac risk evaluation, including blood pressure monitoring, ECG, echocardiography, and cardiac biomarkers in symptomatic cases.

Regarding treatment, beta-blockers can be used to control thyrotoxic symptoms. Additionally, although not routinely indicated, alternative therapies such as triiodothyroacetic acid (TRIAC) have shown promise in reducing TSH and controlling thyrotoxic manifestations in RTHβ, particularly in refractory or symptomatic cases [17]. Unfortunately, the lack of current local availability precludes its use as a therapeutic option in our country.

These cases reaffirm the importance of maintaining a high index of suspicion for RTHβ in patients with discordant TFTs—especially when conventional explanations such as non-compliance or malabsorption are insufficient. Early genetic testing can prevent years of misdiagnosis, avoid unnecessary interventions, and support individualized, patient-centered care. In RTHβ patients with compromised thyroid reserve—whether due to autoimmunity or cytotoxic insult—the usual compensatory mechanisms may fail, necessitating treatment. Lifelong follow-up is essential to anticipate evolving clinical challenges and to tailor treatment across the patient's lifespan.

## 4. Conclusion

The coexistence of RTHβ with autoimmune thyroiditis or amiodarone-induced thyroid dysfunction presents unique diagnostic and therapeutic challenges. The two cases illustrate how overlapping conditions can disrupt the typically compensated state of RTHβ. Accurate diagnosis, longitudinal monitoring, and individualized management—guided by clinical context rather than biochemical thresholds alone—are essential to optimize outcomes in this rare but complex endocrine disorder.

## Figures and Tables

**Table 1:** Serial thyroid function tests and response to levothyroxine dose adjustments in the management of hypothyroidism in a patient with autoimmune thyroiditis and RTHβ.

Laboratory tests and clinical data	Aug/1994	Mar/ 1995	Aug/1996	Jan/1998	Jan/1999	Jul/1999	Oct/2000	Oct/2002	Oct/2003	Oct/2005	Oct/2006	Jun/2017	Jan/2020	Jul/2021	Jan/2022	Apr/2022	Sept/2022	Jul/2023	Jan/2024	Jan/2025
TSH (µIU/mL)	8.75	20.64	5.69	173.3	5.5	23.1	9.3	16.69	4.63	8.18	5.45	3.64-	3.75	4,5	12	27.6	9.78	8.46	17	8.0
TSH (µIU/mL) RR	0.35−5.50	0.35−5.50	0.35−5.50	0.55−4.78	0.55−4.78	0.55–4.78	0.55−4.78	0.55−4.78	0.55–4.78	0.55−4.78	0.55−4.78	0.35−5.5	0.35−5.5	0.35−5.5	0.35−5.5	0.35−5.5	0.35−5.5	0.35−5.5	0.35−5.5	0.35–5.5
FT4 (ng/dL)	1.31	1.16	1.74	0.89	2.5	1.36	1.91	1.62	2.17	1.42	1.9	1.72	2.2	1.92	1.6	1.2	1.78	1.39	1.05	1.7
FT4 RR (ng/dL)	0.65–1.7	0.65–1.71	0.65–1.71	0.89−1.80	0.89−1.80	0.7−1.44	0.7−1.44	0.70−1.44	0.88−1.58	0.88−1.58	0.88−1.58	0.88−1.58	0.88−1.58	0.88−1.58	0.88−1.58	0.88−1.58	0.88−1.58	0.88−1.58	0.88−1.58	0.88−1.58
FT3 (µg/dL)	N.A.	N.A.	5.7	2.5	5.8	3.5	3.17	4.6	N.A.	N.A.	N.A.	N.A.	N.A.	N.A.	3.5	2.2	3.3	3.06	3	3.2
FT3 (µg/dL)	2.3–4.2	2.3–4.2	2.3–4.2	2.3–4.2	2.3–4.2	2.3–4.2	2.3–4.2	2.3–4.2	2.3–4.2	2.3–4.2	2.3–4.2	2.3–4.2	2.3–4.2	2.3–4.2	2.3–4.2	2.3–4.2	2.3–4.2	2.3–4.2	2.3–4.2	2.3–4.2
LT4 (µ/d)	0	0	25	0	88	50	100	125	175	175	200	212	200	175	150	150	175	150	150	175
Symptoms	—	—	—	—	—	—	—	—	—	—	—	Sleep disturbance	Tremorweight loss	—	—	—	—	—	fatigue	—

Abbreviations: d, day; FT3, free triiodothyronine; FT4, free thyroxine; LT4, levothyroxine; RR, reference range; TSH, thyrotropin.

**Table 2:** Serial thyroid function tests and response to amiodarone exposure and levothyroxine dose adjustments in a patient with amiodarone-induced dysfunction and RTHβ.

Laboratory tests and clinical data	Apr/ 2006	Oct/2008	Sept/2012	Aug/2014	May/2016	Jun/2017	May/2016	Jun/2017	Aug/2017	Jan/2018	Jan/2019	Sept/2020	Oct/2020	Jan/2021	Apr/2021	Nov/2021	Jan/2022	Sept/2022	Jan/2023	Aug/2023	Mar/2025
TSH (µIU/mL)	1.55	2.22	1.72	2.73	1.79	4.98	1.79	4.98	7.28	1.07	4.7	11.21	42.7	6.92	9.81	1.25	1.55	1.47	1.49	1.3	1.52
TSH (µIU/mL) RR	0.35−5.50	0.35−5.50	0.35−5.50	0.35−5.50	0.35−5.50	0.35−5.50	0.35−5.50	0.35−5.50	0.35−5.50	0.35−5.50	0.35−5.50	0.35−5.5	0.35−5.5	0.35−5.5	0.35−5.5	0.35−5.5	0.35−5.5	0.35−5.5	0.35−5.5	0.35−5.5	0.35−5.50
FT4 (ng/dL)	1.86	2.02	1.79	N.A.	1.85	2.03	1.85	2.03	1.062	1.33	2.06	1.64	1.06	1.72	1.29	2.08	1.90	1.96	1.74	1.72	1.95
FT4 (ng/dL) RR	0.70–1.48	0.70–1.48	0.70–1.48	0.70–1.48	0.70–1.48	0.70–1.48	0.70–1.48	0.70–1.48	0.70 to 1.48	0.70–1.48	0.70–1.48	0.70–1.48	0.70–1.48	0.70–1.48	0.70–1.48	0.70–1.48	0.70–1.48	0.70–1.48	0.70–1.48	0.70–1.48	0.70–1.48
FT3 (µg/dL)	3.86	3.83	3.30	N.A.	3.49	N.A.	3.49	N.A.	3.67	N.A.	N.A.	N.A.	2.02	4.06	2.94	3.34	3.38	3.57	3.85	3.11	3.48
FT3 (µg/dL) RR	1.75–3.75	1.75–3.75	1.75–3.75	1.75–3.75	1.75–3.75	1.75–3.75	1.75–3.75	1.75–3.75	1.75–3.75	1.75–3.75	1.75–3.75	1.75–3.75	1.75–3.75	1.75–3.75	1.75–3.75	1.75–3.75	1.75–3.75	1.75–3.75	1.75–3.75	1.7–3.75	1.75–3.75
Amiodarone	0	0	0	0	0	0	0	0	^a^200 mg/d	0	0	^b^200 mg/d	0	0	0	0	0	0	0	0	0
LT4 dose (µ/d)	0	0	0	0	0	0	0	0	0	0	0	0	125	125	125	125	88	50	42.8	42.8^c^	0
Symptoms	—	—	Palpitations^1^	—	—	—	—	Palpitations^2^	—	—	Palpitations, fatigue^3^	—	Fatigue with exertional intolerance^4^	—	—	—	—	—	—	—	—

Abbreviations: FT3, free triiodothyronine; FT4, free thyroxine; LT4, levothyroxine; RR, reference; TSH, thyrotropin.

^a^Amiodarone initiated in June 2017 and discontinued in September 2017.

^b^Amiodarone reintroduced in September 2019 and discontinued in September 2020.

^c^Levothyroxine therapy was subsequently withdrawn.

^1^Diagnosis of supraventricular tachyarrhythmia; started on bisoprolol.

^2^Recurrence of the tachyarrhythmia; started on amiodarone.

^3^Admitted to the intensive care unit (ICU) with cardiogenic shock secondary to tachycardia-induced cardiomyopathy.

^4^Readmission for decompensated heart failure.

## Data Availability

The data supporting the findings of this study are available from the corresponding author upon reasonable request.
